# Determinant factors of anaemia among pregnant women attending antenatal care clinic in Northwest Ethiopia

**DOI:** 10.1186/s40794-019-0088-6

**Published:** 2019-07-17

**Authors:** Tadesse Hailu, Simachew Kassa, Bayeh Abera, Wondemagegn Mulu, Ashenafi Genanew

**Affiliations:** 1 Department of Medical Laboratory Science, College of Medicine and Health Sciences, Bahir Dar University, P.O. Box: 79, Bahir Dar City, Ethiopia; 2 Department of Midwifery, College of Medicine and Health Sciences, Bahir Dar University, P.O. Box: 79, Bahir Dar City, Ethiopia; 3 Department of Pharmacy, College of Medicine and Health Sciences, Bahir Dar University, P.O. Box: 79, Bahir Dar City, Ethiopia

**Keywords:** Amaemia, Pregnancy, West Gojjam, Parasitic infection

## Abstract

**Background:**

Anaemia is a low blood haemoglobin concentration and has been shown to be a public health problem affecting both developing and developed countries. Pregnant women are the most vulnerable groups to anaemia due to several factors, including parasitic infection and feeding habits during their pregnancy. The aim of this study was to assess the prevalence and determinant factors of anemia in pregnant women in Northwest Ethiopia.

**Methods:**

A cross-sectional study was conducted among pregnant women from February, 2017 to June, 2017. The data on determinant factors were collected using a structured questionnaire. The hemoglobin level and intestinal parasites were determined using Hemocue HB 201 and formol ether concentration techniques, respectively. Data was entered and analyzed using SPSS version 23 statistical software. Bivariate and multivariate regressions were computed and odds ratio was determined at 95% confidence interval.

**Results:**

The study consists of 743 participants with a median age of 25 years were included. The prevalence of anemia among pregnant women was 79 (10.6%). The prevalence of mild, moderate and severe anaemia were 78 (99.8%), 1 (0.1%) and 1 (0.1%), respectively. Pregnant women of rural dwellers (AOR = 3.72, CI =1.51–9.18), farmer in occupation (AOR = 3.51, CI = 1.75–7.01), and not educated (AOR = 2.25, CI = 1.13–4.48) were significantly associated with increased risk of anemia.

**Conclusion:**

Anaemia is still a problem amongst pregnant women in the study area though much has been done to increase the hemoglobin level during pregnancy. Health education should be given on factors that aggravate anaemia during pregnancy.

## Introduction

Anaemia is a condition of lower red blood cells and haemoglobin (Hgb) level than normal [1]. The prevalence of anemia among pregnant women is estimated to be 38% worldwide, 36.9% in Africa and 23% in Ethiopia [1, 2]. Anaemia can be classified into three catagories, mild, moderate and severe. The Hgb level for each class of anaemia during pregnancy are 10.0–10.9 g/dL (mild), 7–9.9 g/dL (moderate) and < 7 g/dL (severe) [2]. Generally, pregnant women with Hgb level < 11 g/dl are considered to be anaemic [1].

Several factors might contribute to the causes of anemia among pregnant women. For instance, geo-helminth infections during pregnancy may be associated with maternal anaemia. Hookworm is known to be causes of anaemia among pregnant women and hookworm infection mainly aggravates anemia in pregnant women [3]. Infections by geo-helminthes lead to malnutrition, iron deficiency anaemia, and increased vulnerability to other infections in infected pregnant women [4]. The complication may not end up with the maternal anaemia but also causes a complication on the child including; low pregnancy weight gain and intrauterine growth retardation, followed by low birth weight and higher perinatal mortality rates [5].

Pregnant woman that live in rural areas [6], lack of raw vegetables in their diet [7], illnesses [6] and low levels of education [8] are also major determinants of anemia.

Malnutrition is also one of the major causes of anaemia among pregnant women in Ethiopia [9]. Working to improve the nutritional status of pregnant women through supplementation of vitamin A, iron, and iodine is important to minimize the risk of anemia [10].

A lot has been done to minimize the risk of anaemia, but the complication of anaemia is still a problem amongst pregnant women especially in a rural set up. The true prevalence and the determinant factors of anaemia were not well addressed in the study area. Therefore, the present study tried to address the stated information gaps so as to give evidence based action.

## Methods

### Study design, period and area

A cross sectional study was conducted from February, 2017 to June, 2017 among pregnant women in West Gojjam Zone, Northwest Ethiopia. The average elevation of the zone is 2,300 m. The annual temperature of the study area ranges between 16.68 to 37.6 °C.

All pregnant women attending antenatal clinic for the first time during the study period were included in the study. Pregnant women undertaking anthelmintic drugs during the data collection time were excluded from the study. Purposive sampling technique was used to include 743 study subjects. The samples were collected in five woredas as of West Gojjam Zone including: Mecha, Debub Achefer, Bure, Jabi-tihinana and Finot-selam by considering urban and rural settings. One health center was selected in each Woreda based on their laboratory facilities. The sample size in each health institution was 150 which was proportionally allocated by considering the population in the catchment areas.

### Data collection

#### Questionnaires

A structured questionnaire was used to obtain socio-demographic information and determinant factors by interviewing pregnant women. The questionnaire was filled by midwifery health professionals.

#### Haemoglobin determination

The blood samples were collected from a finger prick by blood lancet. Haemoglobin (Hgb) value was determined using a portable Hgb spectrophotometer, Hemocue Hb 201 analyzer (HemoCue, Angelholm, Sweden) and specially designed microcuvette (the Hemocue Hb 201 Microcuvette, Hemocue, Angelholm, Sweden). Then, the Hgb value was then used to assess the status of anaemia. For Hgb the cutoff criterion levels below which indicating aneamia is the WHO cut-off of > 110 g/L for non anemic, 100-109 g/L for mild anemic, 70–90 g/dL moderate and < 70 g/dL for severe anemic for pregnant women [2].

#### Intestinal parasites investigation

Freshly passed stool specimens were collected using a clean plastic cup at the health institutions. The stool cups were labeled with their card number. Laboratory professionals took part in all processes of stool collection and examination. The stool samples were processed for microscopic examination using Formol Ether Concentration Technique (FECT). The stool examination was done in the health institutions laboratory. In FECT, stool sample (0.5 g) was transferred into 10 mL of normal saline in a glass container and mix thoroughly. Two layers of gauze were placed in a funnel and strained the contents into a 15 mL centrifuge tube. Then, 2.5 mL of 10% formaldehyde and 1 mL of ether were added to a test tube. The test tubes were mixed well and centrifuged at 1000 rpm for three minutes. The supernatant was removed and the sediment was mixed well, prepared on two slides one for saline and the other for iodine, and covered with a cover slide and sow under microscope.

#### Data quality assurance

To ensure reliable data collection, training on data collection, laboratory examination and explanation about the study was given before sample collection for midwifery and laboratory personnel. Filled questionnaires were collected after checking for their consistency and completeness. Application of standard procedures during data collection process and accuracy of test results was supervised by the principal investigator. Specimens were cross checked by principal investigators to increase the accuracy of laboratory results. The direct stool microscopy was examined earlier to FECT as soon as the sample arrives. To eliminate observer bias, stool slides were examined independently with two experienced laboratory professionals and 10% of the FECT slides were randomly selected and read by other laboratory professionals as a quality control.

#### Data analysis

Data was entered and analyzed using SPSS version 23 statistical software. The overall magnitude of geo-helminthic infection was calculated using descriptive statistics of the sample through frequencies and cross tabulations. Strength of association between geo-helminthic and determinant factors was calculated by bivariate analysis. The association was analyzed by multivariate logistic regression to avoid confounding effect and calculating the odds ratios (OR) with 95% confidence intervals (CI). In all statistical tests, the differences were considered to be statistically significant if *p*-value less than 0.05.

### Ethical consideration

The proposal was ethically approved by an institutional review board of Bahir Dar University, College of Medicine and Health Science. A written informed consent was obtained from every study participant. Participants tested positive for any parasitic infections got appropriate treatment accordingly from the responsible body.

## Results

### Sociodemographic characteristics of the study subjects

A total of 743 pregnant women took part in this study with a median age of 25 years. The majority (96.2%) of study participants was in the age range 15–35 years. Pregnant women who were rural dwellers and farmers in their occupation accounted 61.2%) and 21.5%, respectively (Table 1).

**Table 1:** Socio demographic characteristics of pregnant women who attended ANC services in West Gojjam Zone, Northwest Ethiopia

Variables		Total [N,%]	Hgb > 11 g/dL	Hgb < 11 g/dL [N,%]	X^2^, p- value
Age in years	15–35	715 [96.2]	646 [90.3]	69 [9.7]	19.20, 0.001
	36–45	28 [3.8]	18 [64.3]	10 [35.7]	
Religion	Christian	736 [99]	657 [89.3]	79 [10.7]	NA
	Muslim	7 [1]	7 [100]	0	
Residence	Rural	455 [61.2]	383 [88.4]	72 [11.6]	7.73, 0.001
	Urban	288 [38.8]	281[97.6]	7 [2.4]	
Woreda of West Gojam	Fenote Selam	150 [20.2]	108 [72]	42 [28]	64.91, 0.001
	Jabitihenane	151 [20.3]	143 [94.7]	8 [5.3]	
	Bure	142 [19.1]	133 [93.7]	9 [6.3]	
	Debub Achefer	150 [20.2]	134 [89.3]	16 [10.7]	
	Mecha	150 [20.2]	146 [97.3	4 [2.7]	
Occupation	Farmer	160 [21.5]	130 [81.3]	30 [18.7]	14.10, 0.001
	Non-Farmer	583 [91.6]	534 [71.9]	49 [8.4]	
Education	Illiterate	372 [50.1]	308 [82.8]	64 [17.2]	17.79, 0.04
	Read & Write	205 [27.6]	203 [99]	2 [1]	
	Primary	88 [11.4]	82 [97.6]	6 [2.4]	
	Junior	9 [1.2]	8 [88.9]	1 [11.1]	
	Secondary	80 [10.8]	75 [93.6]	5 [6.4]	
	> complete	20 [2.7]	19 [95]	1 [5]	
Total		743 [100]	664 [89.4]	79 [10.6]	

### Prevalence of anaemia

The mean Hgb concentration was 12.8 ± 2.97 g/dL with a range of 6 to 17.9 g/dL. The total prevalence of anaemia among pregnant women was 79 (10.6%). The prevalence of mild, moderate and severe anaemia was 9.4, 0.4 and 0.1%, respectively. Mild anaemia had high prevalence 77 (97.5%) among anaemic pregnant women (Table 2).

**Table 2:** The level of anaemia and intestinal parasitic infection among pregnant women in West Gojjam, Northwest Ethiopia

Types of IP			Level of anaemia				
		Total [N,%]	Non-anaemic [N,%]	Mild [N,%]	Moderate [N,%]	Severe [N,%]	Total anaemia [N,%]
Hookworm	Pos	138 [18.6]	99 [71.7]	39 [28.3]	0	0	39 [5.2]
	Neg	605 [81.4]	565 [93.4]	38 [6.2]	1 [0.2]	1 [0.2]	40 [5.4]
*G. lamblia*	Pos	53 [7.1]	30 [56.6]	23 [43.4]	0	0	23 [3.1]
	Neg	690 [93.2]	634 [91.8]	54 [7.8]	1[0.2]	1 [0.2]	56 [7.5]
*E. histolytica*	Pos	113 [15.2]	85 [75.2]	26 [23]	1 [0.9]	1 [0.9]	28 [3.8]
	Neg	630 [84.8]	579 [91.9]	51 [8.1]	0	0	51 [6.8]
All Parasite	Pos	278 [37.4]	222 [79.9]	55 [19.8]	1 [0.3]	0	56 [7.5]
	Neg	465 [62.6]	442 [95.1]	20 [4.3]	2 [0.4]	1 [0.2]	23 [3.1]
Total		743 [100]	743 [100]	664 [89.4]	75 [10.1]	3 [0.4]	1 [0.1]

*Pos* Positive; *Neg* Negative

### Prevalence of intestinal parasitosis

The overall prevalence of intestinal parasitosis among pregnant women was 276 (37.1%). Hookworm has the highest prevalence 138 (50%) among the parasitic infected pregnant women followed by *E. histolytica* 113 (40.9%) and *G. lamblia* 53 (19.2%) (Table 2). The prevalence of anaemia among hookworm, *E.histolytica/dipar and G. lamblia* infected pregnant women were 39 (28.3%), 28 (24.8%) and 23 (43.4%), respectively (Table 2).

### Obstetrics and medical condition of study participants

There were 509 (68.5%) multi gravid and 420 (56.5%) second trimester pregnancy study participants. The prevalence of anaemia among Multi gravid, one parity and third trimester were 57 (11.2%), 21 (13.2%) and 31 (13.4%), respectively. The prevalence of anaemia was 77.8 and 18.1% among pregnant women who had a previous malaria attack and history of intestinal parasitic infection, respectively (Table 3).

**Table 3:** Obstetrical & Medical characteristics of pregnant women who attended ANC service in West Gojjam Zone, Northwest Ethiopia

Variables		Frequency [N,%]	Hgb > 11 g/dL[N,%]	Hgb < 11 g/dL [N,%]	X^2^*, p-value*
Gravidity	Primigravida	234 [31.5]	212 [90.6]	22 [9.4]	0.55, 0.52
	Multi gravid	509 [68.5]	457 [88.8]	57 [11.2]	
Parity	No	241 [32.4]	218 [90.5]	23 [9.5]	1.48, 0.69
	1	159 [21.4]	138 [86.8]	21 [13.2]	
	2–3	224 [30.2]	201 [89.7]	23 [10.3]	
	> 4	119 [16]	107 [89.9]	12 [10.1]	
Trimester	First	142 [19]	135 [95.1]	7 [4.9]	7.22, 0.30
	Second	326 [44]	285 [87.4]	41 [12.6]	
	Third	275 [37]	244 [88.7]	31 [11.3]	
History of malaria infection	Yes	9 [1.2]	2 [22.2]	7 [77.8]	43.22, 0.00
	No	734 [98.8]	662 [90.2]	72 [9.8]	
History intestinal parasite infection	Yes	276 [37.1]	226 [18.9]	50 [18.1]	25.88, 0.00
	No	467 [62.9]	428 [93.8]	29 [6.2]	
History of previous illness	Yes	115 [15.5]	72 [62.6]	43 [37.4]	24.14, 0.00
	No	628 [84.5]	592 [94.3]	36 [5.7]	

### Determinant factors for anaemia in pregnancy

Pregnant women in rural areas were 3.72 times more likely to be anaemic than urban dwellers (AOR = 3.72 [95%CI:1.51–9.18]). Likewise, farmer pregnant women were 3.51 times more likely to be anaemic with non-farmer ones (AOR = 3.51 [95%CI:1.75–7.01]). In the same way, non previously medically ill pregnant women were 85% less likely to be anaemic than previously medically ill ones (AOR = 0.15[95%CI:0.08–0.28]. Pregnant women who did not eat raw vegetables were 8.94 times more likely to become anemic than pregnant women who ate raw vegetables (AOR = 8.94 [95%C:2.86–10.55]). Pregnant women who didn’t eat meat were 11.49 times more likely to be anaemic than those who ate meat (AOR = 11.49 [95% CI:2.51–12.53]) (Table 4). Illiterate Pregnant women were 2.25 times more likely become anaemic than not the illiterate once (AOR = 2.25 [95%CI:1.13–4.48]). Those pregnant women infected with hookworm parasites were 3.81 times more likely to develop anaemia than pregnant women who were not infected with hookworm parasites (AOR = 3.81 [95%CI,2.06–7.06]) (Table 4).

**Table 4:** Multivariate analysis showing the Determinant factors of anaemia among pregnant women in West Gojjam zone, 2017

Variables		Anemia present		COR[95%CI]	AOR[95%CI]	*P* value
		Yes (N)	No (N)			
Address	Rural	72	383	7.55[3.42–.16.65]	3.72[1.51–9.18]	0.004
	Urban	7	281	1	1	
Occupation	Farmer	30	130	2.52[1.54–4.12]	3.51[1.75–7.01]	0.001
	Non Farmer	49	534	1	1	
Eating raw vegetables	No	75	490	6.66[2.40–18.47]	8.94[2.86–10.53]	0.001
	Yes	4	174	1	1	
Age	15–35	69	646	1		
	36–45	10	18	5.20[2.31–11.71	4.46[1.57–12.69]	0.005
Eating meat	No	76	568	4.28[1.32–13.88]	11.49[2.51–12.53]	0.002
	Yes	3	96	1		
Previous illness	Yes	43	72	9.82[5.92–16.29]	0.15[0.08–0.28]	0.001
	No	36	592	1	1	
Educational status	Illiterate	64	308	4.93[2.75–8.83]	2.25[1.13–4.48]	0.02
	Not illiterate	15	356	1	1	
Hookworm infection	Yes	41	97	6.31[3.86–10.31]	3.81[2.06–7.06]	0.001
	No	38	567	1	1	

## Discussion

Anaemia is an important complication during pregnancy, especially in a rural set up. The impacts of anaemia rest upon not only on the health of pregnant women, but also on her offspring. In the present study, the overall magnitude of anaemia among pregnant women was 10.6%. This result was comparable with previous studies done in Amhara Regional State (15.89%) [11], Gondar city (16.6%) [12] and Iran (13.1%) [13]. The current prevalence was also considerably lower than previous reports from Jimma, Southern Ethiopia (38.2%) [14], Tigray (36.1%) [15], Southern Ethiopia (23.2%) [16], Jordan (34.7%) [17], Vietnam (43.2%) [18] , and Southeastern Nigeria (76.9%) [19]. The possible explanation for the difference might be geographical variation of factors across different areas. The lower prevalence in the present study could be attributed to gradual improvement of lifestyle and living standards and health seeking behavior by the effort of government to achieve the sustainable development goal aimed to reduce the maternal mortality.

In the present study, most of the anaemic pregnant women had mild anaemia. This finding was comparable with a result obtained previously in Northwest Ethiopia [20, 21], Southeast Ethiopia [22] and Pakistan [23], but different from other reports [24]. The difference may be due to a regular supply of iron and folic acid supplimentation and anti-helminthic drugs in the present study.

Parasitic infection has a devastating effect on the level of Hgb and causes anaemia since they affect iron absorption by the intestine and consumes the red blood cells [25]. In the present study, pregnant women who had previous medical illness, malaria infection and intestinal parasitic infection were more likely to become anaemic. This was consistent with previously conducted in Southern Ethiopia [16], Ghana [25], Nigeria [26], and Venezuela [27].

The level of Hgb may be varied during the course of pregnancy by several obstetric factors. In the present study, the level of Hgb is varied by gravidity, parity and trimesters. This result was similar with different findings conducted previously in Southern Ethiopia [16], Southeast Ethiopia [22], Ghana [25] and Iran [28].

In the current study, pregnant women living in rural areas were 3.72 times more likely to be anaemic as compared to those living in urban areas. Similar results were reported by studies conducted in Gondar [12], and southwest Ethiopia [22]. The possible reason might be due to low socioeconomic status, lack of adequate information about nutrition during pregnancy and accessibility to health care facilities and illiteracy.

Lack of awareness about anaemia and its impact during pregnancy may be a major factor becoming anaemic of pregnant women. In the present study, farmer pregnant women were 3.51 times more likely to be anaemic with that of non farmer pregnant women. A similar result was obtained in a study done in India [29]. Pregnant women who did not get formal education were 2.25 times more likely for anaemic than from those who got formal education. Similar findings were reported in India [19] and China [30].

Pregnant women who did not eat raw vegetables were 8.94 times more likely to have anemia compared to pregnant women who eat raw vegetables. This finding is consistent with different parts of Ethiopia [20, 31, 32]. The possible reason might be due to poor dietary diversity which leads to a deficiency of minerals and vitamins which may increase bio-availability of iron. Pregnancy is the most nutritionally demanding period in a woman’s life. Consequently, pregnant women are advised to eat a more diversified diet than usual.

Pregnant women who didn’t eat meat were 11.49 times more likely to be anaemic than those who eat meat. This finding was consistent with other studies in which pregnant women conducted in Ethiopia who ate red meat [31, 32] and Pakistan [23]. The increased concentration of Hgb is with the fact that red meat is an important source of heme iron [33] which is a major component of red blood cells.

Soil transmitted helminths like hookworm infection causes anaemia during pregnancy. In the current study, pregnant women infected with hookworm parasites were 3.81 times more anaemic than non hookworm infected pregnant women. This finding was consistent with previous studies conducted among pregnant women in East Wollega, Oromia, Ethiopia [34] and Northwest Ethiopia [35]. This study did not include all health institutions due to the absence of full antenatal care service including detection of intestinal parasitosis and determination of the Hgb level.

## Conclusion

Anaemia is one of the major complications during pregnancy. Most pregnant women had mild anaemia in the present study. Factors related to obstetric conditions, nutrition, parasitic infection were the main risk factors. Therefore, awareness should be built among pregnant women targeting on their feeding habits, parasitic infection and hygienic conditions to minimize the outcomes of anaemia during pregnancy.

## Data Availability

To generate findings of this particular study, data was collected and analyzed.

## Abbreviations

FECT: Formol Ether Concentration Technique
Hgb: Haemoglobin
